# A comparison of DNA sequencing and the hydrolysis probe analysis (TaqMan assay) for knockdown resistance (*kdr*) mutations in *Anopheles gambiae *from the Republic of the Congo

**DOI:** 10.1186/1475-2875-9-278

**Published:** 2010-10-12

**Authors:** Kwang Shik Choi, Belinda L Spillings, Maureen Coetzee, Richard H Hunt, Lizette L Koekemoer

**Affiliations:** 1 Vector Control Reference Unit, National Institute for Communicable Diseases of the National Health Laboratory Service, Johannesburg, South Africa; 2 Malaria Entomology Research Unit, School of Pathology, Faculty of Health Sciences, University of the Witwatersrand, Johannesburg, South Africa; 3 School of Animal, Plant and Environmental Sciences, University of the Witwatersrand, Johannesburg, South Africa

## Abstract

**Background:**

Knockdown resistance (*kdr*) caused by a single base pair mutation in the sodium channel gene is strongly associated with pyrethroid insecticide resistance in *Anopheles gambiae *in West-Central Africa. Recently, various molecular techniques have been developed to screen for the presence of the *kdr *mutations in vector populations with varying levels of accuracy. In this study, the results of the hydrolysis probe analysis for detecting the *kdr *mutations in *An. gambiae *s.s. from the Republic of the Congo were compared with DNA sequence analysis.

**Methods:**

A total of 52 pyrethroid and DDT resistant *An. gambiae *from Pointe-Noire (Congo-Brazzaville) were tested for detection of the two *kdr *mutations (*kdr*-e and *kdr*-w) that are known to occur in this species. Results from the hydrolysis probe analysis were compared to DNA sequencing to verify the accuracy of the probe analysis for this vector population.

**Results:**

Fifty-one specimens were found to be *An. gambiae *S-form and one was a M/S hybrid. DNA sequencing revealed that more than half of the specimens (55.8%) carried both the *kdr*-e and *kdr*-w resistance mutations, seven specimens (13.5%) were homozygous for the *kdr*-e mutation, and 14 specimens (26.9%) were homozygous for the *kdr*-w mutation. A single individual was genotyped as heterozygous *kdr*-e mutation (1.9%) only and another as heterozygous *kdr*-w mutation (1.9%) only. Analysis using hydrolysis probe analysis, without adjustment of the allelic discrimination axes on the scatter plots, revealed six specimens (11.5%) carrying both mutations, 30 specimens (57.8%) as homozygous *kdr*-w, six specimens (11.5%) homozygous for the *kdr*-e mutation, one specimen (1.9%) heterozygous for the *kdr*-w mutation and one specimen (1.9%) present in wild type form. Eight of the specimens (15.4%) could not be identified using unadjusted hydrolysis probe analysis values. No heterozygous *kdr*-e mutations were scored when adjustment for the allelic discrimination axes was omitted. However, when the axes on the scatter plots were adjusted the results were consistent with those of the DNA sequence analysis, barring two individuals that were mis-scored in the hydrolysis probe analysis.

**Conclusion:**

Both the *kdr*-e and *kdr*-w mutations were abundant in *An. gambiae *S-form from Pointe-Noire. The hydrolysis probe analysis can lead to misleading results if adjustment to allelic discrimination axes is not investigated. This is mainly relevant when both *kdr*-e and *kdr*-w are present in a population in a high frequency. This report highlights the importance of concurrent screening for both mutations. Therefore, performing routine assay protocols blindly can result in the misinterpretation of results. Although hydrolysis probe analysis of *kdr *is still held as the gold standard assay, this paper highlights the importance of *kdr *mutation confirmation via sequencing especially in regions where *kdr *frequency has never been reported before or where both the *kdr*-e and *kdr*-w mutations are present simultaneously.

## Background

One of the main strategies for malaria vector control is the use of pyrethroid insecticides for indoor residual spraying (IRS) and for the impregnation of long-lasting bed nets (LLINs). Reports of insecticide resistance in the major African malaria vectors have increased dramatically over the past few years [1-13]. The monitoring of the presence and development of insecticide resistance in malaria vector populations is essential for understanding resistance mechanisms and for the downstream implementation of insecticide resistance management plans.

Pyrethroids and organochlorines (eg. DDT) target the para-type sodium channel gene in the insect nervous system [14,15]. Cross resistance between these two classes of insecticides is reported when mutations in the target site results in insensitivity to these chemicals [16-20].

Two different single nucleotide mutations in the segment 6 of domain II region of the *para*-type sodium channel gene have been reported in *An. gambiae*. Martinez-Torres *et al *[21] reported that the *kdr *mutation in *An. gambiae *from West Africa leads to the substitution of a leucine (TTA) for phenylalanine (TTT) (L1014F, *kdr*-w). Ranson *et al *[22] reported a second *kdr *mutation in the same amino acid position in *An. gambiae *from Kenya. This mutation causes a substitution from leucine (TTA) to serine (TCA) (L1014 S, *kdr*-e) and is referred to as the East African mutation or *kdr*-e. More recently studies have reported heterozygous substitutions for both *kdr *mutations in *An. gambiae *from Cameroon (8% [6]; 1.7% [7]; 16.1% [8]), Gabon (55.7%) [9] and Uganda (1.4%) [10].

Currently, there are several different assays available for screening the DNA substitutions related to the *kdr *mutations in *An. gambiae*. The most commonly used method for detecting the *kdr*-e and *kdr*-w mutations in *An. gambiae *is carried out using two different allele-specific polymerase chain reaction assays (AS-PCR) [21,22]. However, these assays can lead to inaccurate results due to a single nucleotide polymorphism mismatch at the 3'-end of the primer. In order to address the unreliability of this assay, several recent techniques have been developed such as: PCR sequence specific oligonucleotide probe assay (PCR-Dot Blot) [23], Heated Oligonucleotide Ligation Assay (HOLA) [24], Fluorescence Resonance Energy Transfer (FRET)/Melt Curve analysis [10], PCR elongation with fluorescence [25], Sequence Specific Oligonucleotide Probe Enzyme-Linked ImmunoSorbent Assay (SSOP-ELISA) [26], hydrolysis probe analysis [27], Primer Introduced Restriction Analysis-PCR assay (PIRA-PCR) [28], multiplex PIRA-PCR assay (mPIRA-PCR) [29] and pyrosequencing [30]. With regard to specificity and sensitivity for detecting the *kdr *mutations, the hydrolysis probe analysis can be considered to be one of the best [27]. This study shows that this assay remains one of the best assays to use but that both *kdr *mutations should be screened concurrently. Changing default setting on machine may further enhance the accuracy of results.

## Methods

### Mosquito collections and identification

Fifty two mosquitoes were collected indoors in Pointe-Noire, Republic of the Congo (Congo-Brazzaville) (4°48'38''S, 11°53'9''E) in April 2009 as part of a survey for insecticide resistance. The specimens were identified as *An. gambiae *s.l. using the keys of Gillies and Coetzee [31]. DNA was extracted from either the abdomen or parts of mosquitoes according to Collins *et al *[32]. DNA concentration was measured using a NanoDrop spectrophotometer (NanoDrop Technologies) and the amount of DNA varied between 5.1 and 429.9 ng/μL. The method of Scott *et al *[33] was used to identify the specimens to species level and M/S status was determined using a modified method from Favia *et al *[34].

### Hydrolysis probe analysis

The protocol of Bass *et al *[27] was used with minor modifications. PCR was performed using a CFX96™Real-Time system (Bio-Rad). Two standard oligonucleotides (Inqaba Biotech) and three minor groove binding (MGB) probes (Applied Biosystems) were used. Primers *kdr*-Forward (5'-CATTTTTCTTGGCCACTGTAGTGAT-3') and *kdr*-Reverse (5'-CGATCTTGGTCCATGTTAATTTGCA-3') were used for binding the flanking region of both *kdr *mutation sites in the sodium channel gene. The probe WT (5'-CTTACGACTAAATTTC-3') was labelled with VIC for the detection of the wildtype allele and the probes *kdr*E (5'-ACGACTGAATTTC-3') and *kdr*W (5'-ACGACAAAATTTC-3') were labelled with 6-FAM for detection of the *kdr*-e and *kdr*-w resistant alleles respectively. The two standard primers (*kdr*-Forward and *kdr*-Reverse) and the WT probe were used either in one reaction with the *kdr*E probe for detecting *kdr*-e or in another reaction with the *kdr*W probe for detecting *kdr*-w. The 20 μL PCR reaction contained 1 μL of the genomic DNA of an individual mosquito, 10 μL of IQ™Supermix (Bio-Rad), 0.8 μM of each primer and 0.4 μM of each probe. The PCR cycling conditions were as follows: an initial denaturation at 95°C for 10 minute, followed by 40 cycles of 95°C for 10 seconds and 60°C for 45 seconds. The increase in VIC and FAM fluorescence was monitored in real time by detecting fluorescence of VIC (560-580 nm detection) and FAM (515-530 nm detection) channels for each dye respectively. The assay was repeated at least twice to ensure that experimental error was limited.

### DNA sequencing

Genotypes of individuals were confirmed by DNA sequencing. A segment of the *kdr*-e and *kdr*-w mutations region in the *para*-type sodium channel gene from 52 individuals was amplified using Agd1 (5'-ATAGATTCCCCGACCATG-3') and Agd2 (5'-AGACAAGGATGATGAACC-3') primers [21]. A total volume of 50 μL for each reaction contained average 91.3 ng genomic DNA of an individual mosquito, 1× PCR Buffer, 1.5 mM MgCl_2_, 0.2 mM of each dNTP, 0.4 μM of each primer, and 1 unit of *Taq *DNA polymerase. The cycling conditions were as follows: a 2 minute 94 °C denaturation step; 40 cycles of 30 seconds at 94 °C, 30 seconds at 50°C and 30 seconds at 72°C; final extension for 5 minutes at 72°C. The PCR products were electrophoresed on 1.5% agarose gel containing 0.5 μg/mL ethidium bromide. Direct PCR sequencing was performed by MACROGEN (Seoul, Korea) for both strands using the Adg1 and Agd2 primers. The results were aligned and analysed using Clustal X and Bioedit.

## Results

Of the 52 specimens used, 51 were *An. gambiae *S-forms and one individual was identified as a hybrid M/S. This was confirmed using DNA sequencing as well as the method of Fanello *et al *[35]. The hybrid M/S specimen carried both *kdr *mutations. No M-form specimens were identified in this study. Comparison between the hydrolysis probe analysis (default setting for the allelic discrimination axes) and DNA sequencing revealed that only 51.9% for the *kdr*-e and 86.5% for the *kdr*-w gave similar results (Table 1). For this reason the axes used for allelic discrimination were adjusted and data re-analysed.

**Table 1:** Comparison of the frequency of the *kdr*-e and *kdr*-w mutations using DNA sequence, and hydrolysis probe analysis with and without adjustment of the allelic discrimination axes.

	Genotypes					
Detection method	Ser-Phe	Ser-Ser	Phe-Phe	Ser-Leu	Phe-Leu	No ID
DNA sequence	29 (55.8%)	7 (13.5%)	14 (26.9%)	1 (1.9%)	1 (1.9%)	0 (0%)
Hydrolysis probe analysis without adjustment	6 (11.5%)	6 (11.5%)	30 (57.8%)	1 (1.9%)	1 (1.9%)	8 (15.4%)
Hydrolysis probe analysis with adjustment	27 (51.9%)	8 (15.4%)	15 (28.9%)	1 (1.9%)	1 (1.9%)	0 (0%)

A total samples were 52 specimens. Ser = L1014 S (*kdr*-e); Phe = L1014F (*kdr*-w); Leu = Wild type; No ID = failed to amplify

### Hydrolysis probe analysis

**Figure 1:**
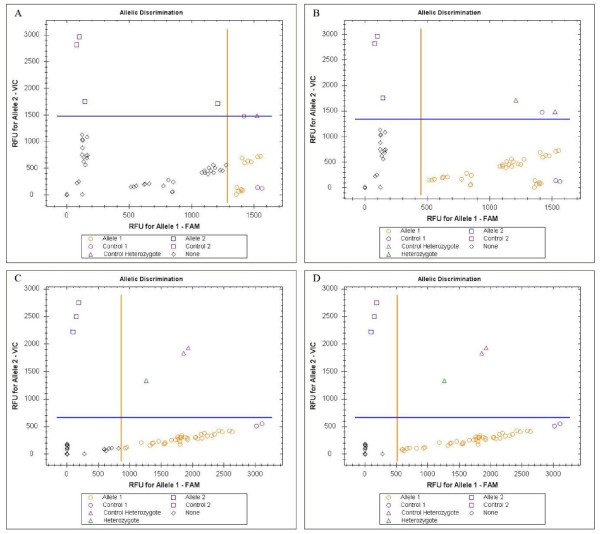
**Scatter plot analysis of hydrolysis probe analysis fluorescence results for the *kdr*-e and *kdr*-w assays before and after adjustment of the allelic discrimination axes**. A) The default allelic discrimination axes for the *kdr*-e mutation assay. B) After adjusting the allelic discrimination axes for the *kdr*-e mutation assay were shown. C) The default allelic discrimination axes for the *kdr*-w mutation assay. D) After adjusting the allelic discrimination axis for the FAM only.

The first specific probe for the wild type allele was labelled with VIC and the second specific probe for the *kdr*-e or *kdr*-w alleles was labelled with FAM. It was essential to determine the standard values of fluorescence for scoring the genotypes in order to produce accurate results. The results from the hydrolysis probe analysis in this study were only consistent with the results from the DNA sequence when the allelic discrimination axes were adjusted (10% and 65% for the *kdr*-e allele susceptible and resistant probes with VIC and FAM respectively and 35% for the *kdr*-w resistant probe with FAM) from the original default axes as determined by the hydrolysis probe analysis programme were performed (Figure 1). No adjustment was needed for the wild type susceptible probe when it was run in the *kdr*-w reaction. One sample was identified as RR using *kdr*-w probe, while this specimen gave a failed reaction when using *kdr*-e analysis upon sequencing. This sample was shown to be homozygous for both *kdr*-e and *kdr*-w mutations. A second sample failed in *kdr*-w analysis, but was identified as RR using the *kdr*-e analysis. This specimen was also sequenced and shown as homozygous for both *kdr*-e and *kdr*-w. Therefore, 2/52 or 4% of samples resulted in discrepancies between the sequence and hydrolysis probe results. Adjustment of the allelic discrimination axes did not alter this result.

### DNA sequencing

The region for the *kdr *mutations in the sodium channel gene sequenced includes 293 bp of coding region. The results were aligned with each of the allele sequences from GenBank. The data showed 55.8% (29/52) of specimens carried both the *kdr*-e and *kdr*-w mutations in heterozygote form. Seven specimens were homozygous for the *kdr*-e mutation and 14 homozygous for the *kdr*-w mutation. There was only one specimen heterozygous for the *kdr*-e mutation and one other that was heterozygous for the *kdr*-w mutation. Sequence analysis did not reveal any homozygous susceptible specimens for both *kdr *mutations i.e. the wild type form.

## Discussion

Most of the specimens collected in Pointe-Noire were *An. gambiae *S-form (98.1%). Unexpectedly, one specimen was identified as a hybrid M/S whilst no *An. gambiae *M-form were collected in this study area. Koekemoer *et al *(Multiple insecticide resistance in *Anopheles gambiae *(Diptera: Culicidae) from Pointe Noire, Republic of the Congo, submitted) published additional data from this area from 343 specimens. This was the only hybrid identified from large sample analysis and additional genetic studies would need to be concluded to explain the presence of the hybrid in the rare presence of *An. gambiae *M-form (0.3%) by performing analysis of large sample size. The presence of the *kdr *mutations within this study cohort was unexpectedly high (100% of sequenced samples; 96% of hydrolysis probe analysis samples). Both *kdr*-e and *kdr*-w resistance mutations occurred simultaneously in 29 (55.8%) out of 52 specimens. The frequencies of the homozygous *kdr*-e and *kdr*-w single mutations were 7 (13.5%) and 14 specimens (26.9%) respectively. There was one specimen heterozygous for the *kdr*-e (1.9%) and another heterozygous for the *kdr*-w (1.9%) mutations. Although recent studies reported that the frequencies of the both mutations present at the same time were much lower (1.7-16.1% in Cameroon [6-8] and 1.4% in Uganda [10]) than the present study, Pinto *et al *[9] reported that all *An. gambiae *specimens collected in Gabon in 2000 carried the *kdr*-e or *kdr*-w mutations and 55.7% of the co-occurring both mutations. A recent study has reported that the *An. gambiae *population from Congo was resistant to DDT (100%), deltamethrin (26%), dieldrin (69%) and bendiocarb (3%) (Koekemoer *et al*: Multiple insecticide resistance in *Anopheles gambiae *(Diptera: Culicidae) from Pointe Noire, Republic of the Congo, submitted). This study also suggested that both *kdr *mutations in *An. gambiae *are widespread in the region.

Although DNA sequencing remains the most accurate method for detecting the presence of *kdr *mutations, it is much more time consuming and expensive (> US$10 per sample for sequencing in both directions) than other assays. Bass *et al *[27] reported that the hydrolysis probe analysis is the most sensitive and specific assay for detecting *kdr *mutations (5.2% of failed reactions and 0% of mis-scores) when compared to AS-PCR, HRM, HOLA, SSOP-ELISA and PCR-Dot Blot assays even though the cost of the assay (US$1.72 per sample) is higher than some of the other methods (US$0.62 - US$ 1.74 per sample). In this study, 4% (2/52) samples gave different results between the hydrolysis probe and sequencing assays due to a failed reaction of either the *kdr*-e or *kdr*-w PCR.

Results from this study recommend that the allelic discrimination axes used for detecting both mutations simultaneously can be adjusted to increase sensitivity of the assay. This is especially true in those populations carrying both mutations at high frequency. However, this decision should be made cautiously and sequencing should be used to validate the adjustments made.

The accuracy of the assay could fluctuate due to the determination of the allelic discrimination values for the axes as well as due to other variable running conditions. A number of conditions such as quality and quantity of DNA templates, master mixes, PCR assay performers, the positioning of the allelic discrimination axes and other conditions might affect *kdr *genotyping. The best method for genotyping seems to be end point analysis as well as intensity of fluorescence, although two out of fifty two samples in this study could not be genotyped using these values due to misleading RFU values, even after adjustment of the default allelic discrimination axes.

## Conclusions

Various new assays have been developed to screen *kdr *mutations in *An. gambiae *and the hydrolysis probe analysis is a preferred method. As this assay becomes more widely used in African laboratories it is important to emphasize that accurate genotyping in *An. gambiae *will rely on concurrent testing of both *kdr*-e and *kdr*-w mutations. In addition, this should be noted that default settings might have to be adjusted to enhance results.

## Conflict of interests

The authors declare that they have no competing interests.

## Authors' contributions

KSC carried out identification of species and molecular forms of *An. gambiae*, *kdr*-e and *kdr*-w assays and DNA sequence, analysed the data and drafted the manuscript. BLS contributed to the design of this study, carried out identification of species, repetition of both *kdr *mutation assays, helped analyse the data and draft the manuscript. RHH collected all specimens from Pointe-Noire, identified species morphologically and helped draft the manuscript. LLK and MC assisted in data analysis and drafting of the manuscript.

All authors have read and approved the final manuscript.
